# Impact of an informed choice invitation on uptake of screening for diabetes in primary care (DICISION): trial protocol

**DOI:** 10.1186/1471-2458-9-63

**Published:** 2009-02-20

**Authors:** Eleanor Mann, A Toby Prevost, Simon Griffin, Ian Kellar, Stephen Sutton, Michael Parker, Simon Sanderson, Ann Louise Kinmonth, Theresa M Marteau

**Affiliations:** 1 Psychology Department (at Guy's), Health Psychology Section, Psychology and Genetics Research Group, 5th Floor Bermondsey Wing, Guy's Campus, London, SE1 9RT, UK; 2 Department of Public Health and Primary Care, University of Cambridge, Forvie Site, Robinson Way, Cambridge, CB2 0SR, UK; 3 MRC Epidemiology Unit, Institute of Metabolic Science, Box 285, Addenbrooke's Hospital, Hills Road, Cambridge, CB2 0QQ, UK; 4 The Ethox Centre, Division of Public Health and Primary Health Care, University of Oxford, Badenoch Building, Old Road Campus, Headington, Oxford, OX3 7LF, UK

## Abstract

**Background:**

Screening invitations have traditionally been brief, providing information only about population benefits. Presenting information about the limited individual benefits and potential harms of screening to inform choice may reduce attendance, particularly in the more socially deprived. At the same time, amongst those who attend, it might increase motivation to change behavior to reduce risks. This trial assesses the impact on attendance and motivation to change behavior of an invitation that facilitates informed choices about participating in diabetes screening in general practice. Three hypotheses are tested:

1. Attendance at screening for diabetes is lower following an informed choice compared with a standard invitation.

2. There is an interaction between the type of invitation and social deprivation: attendance following an informed choice compared with a standard invitation is lower in those who are more rather than less socially deprived.

3. Amongst those who attend for screening, intentions to change behavior to reduce risks of complications in those subsequently diagnosed with diabetes are stronger following an informed choice invitation compared with a standard invitation.

**Method/Design:**

1500 people aged 40–69 years without known diabetes but at high risk are identified from four general practice registers in the east of England. 1200 participants are randomized by households to receive one of two invitations to attend for diabetes screening at their general practices. The intervention invitation is designed to facilitate informed choices, and comprises detailed information and a decision aid. A comparison invitation is based on those currently in use. Screening involves a finger-prick blood glucose test. The primary outcome is attendance for diabetes screening. The secondary outcome is intention to change health related behaviors in those attenders diagnosed with diabetes. A sample size of 1200 ensures 90% power to detect a 10% difference in attendance between arms, and in an estimated 780 attenders, 80% power to detect a 0.2 sd difference in intention between arms.

**Discussion:**

The DICISION trial is a rigorous pragmatic denominator based clinical trial of an informed choice invitation to diabetes screening, which addresses some key limitations of previous trials.

**Trial registration:**

Current Controlled Trials ISRCTN73125647

## Background

Invitations to attend for screening traditionally provide information about its population benefits and aim to achieve high rates of uptake [1]. They do not usually refer to the likelihood of health benefits for the individual, which are low [2], nor to the possibility of adverse effects. This neglect reflects a greater concern with potential public health benefits than with individual autonomy. There has, however, been a policy change in the UK and elsewhere [3,4] towards a view that participation in screening programmes should reflect individual choices informed about both the nature and frequency of possible individual benefits and harms of screening. Invitations to participate in screening do not routinely reflect this policy change. This may reflect a reluctance of those organizing screening programmes to implement a policy change that may privilege concern for informed choice to the neglect of achieving the public health benefits of screening [5]. Giving information about the type and frequencies of individual benefits and burdens could deter some people from participating in screening programmes [6-9]. We therefore test the hypothesis that facilitating informed choice results in lower screening attendance than when informed choices are not facilitated.

A further concern is that invitations that support informed choice may reduce uptake differentially across social groups resulting in lower uptake amongst the more socially deprived. These groups are also likely to be those in poorest health and hence those in whom the benefits of screening might be greatest [10]. Given that participation in screening programmes is already lower in these groups [11], an informed choice policy could increase the health gap between socially more and less advantaged groups in one of two ways. Information on the limitations of screening may have greater impact upon the more socially deprived because they are less aware of such limitations [12-14]. Information about the possible longer term benefits and more immediate harms of screening may also be more demotivating among this group which is known to be oriented more towards the present than to the future, both generally and in relation to their health [15,16]. Indeed, one of the few studies to examine the impact of risk information across social groups found that a risk counseling intervention designed to increase attendance for mammography had no impact on those with high levels of education but decreased attendance in those with lower levels [17]. Consequently, we predict that an informed choice invitation will differentially affect attendance among individuals who are more socially deprived compared with those who are less socially deprived.

Informed choice invitations attempt to enhance individual autonomy. This may lead to increased motivation to reduce identified risk among those accepting the invitation for screening [18-22]. According to Self Determination Theory [22] autonomous decisions are more intrinsically motivated; that is, the individual has greater personal interest in the behavior, thus the decision is more likely to lead to action [23]. In the case of diabetes, the most effective way of reducing risk is by changing behavior (improving diet, increasing physical activity, taking medication) [24,25]. Therefore, we predict stronger intentions to engage in these risk reducing behaviors in those attending for diabetes screening after receiving an informed choice invitation than in those who received a standard invitation.

The ethical dilemma central to the proposed research concerns the potential conflict between two fundamental moral principles guiding health care practice and policy: on the one hand, patient-centered practice, which privileges the principle of respect for individual autonomy; and on the other, the promotion of public health benefits, which privileges achieving the greatest overall benefit. The current trial examines whether such an ethical conflict follows the implementation of an informed choice policy in the context of screening.

### Achieving informed choices in screening

Informed choices or decisions can be considered to have two theoretical core characteristics: first, they should be informed by best current evidence; and second, they should reflect the decision-maker's values [26,27]. In making an informed choice people are neither deceived nor coerced [28]. Individuals first need good quality information that can be read and understood across a wide range of literacy as a necessary, although not sufficient, basis for an informed choice.

Decision aids aimed at conveying written information and structuring the decision-making process can be used to facilitate informed choices about a range of health interventions including screening. The few published evaluations of self-administered decision aids provide some evidence that knowing about and evaluating the personal importance of the benefits and risks associated with a particular choice can help individuals to clarify their values. This improves the quality of their subsequent decisions [29]. Thus, for example, the use of a decision aid supporting women's decisions to use hormone replacement therapy resulted in more accurate perceptions of breast cancer risks, greater confidence to make a decision and more satisfaction with the decision made [30]. However, the evidence is limited and mixed. Four of the 10 studies reviewed by O'Connor and colleagues [29] found significant improvements in decision quality, as measured by the decisional conflict scale [31]; the remaining six studies found no significant effects of a decision aid in improving the decision making process.

The impact of decision aids on screening uptake has also been mixed and small. Krist, Woolf, Johnson, & Kerns [32] found requests for prostate cancer screening tests were lower in those using a decision aid, whereas Mathieu and colleagues [33] reported no difference in breast cancer screening uptake in women using a decision aid compared to those not using the aid. Similarly, Trevena, Irwig, & Barratt [34] found no impact of a decision aid on rates of self reported use of colorectal screening kits.

Differences in findings between studies may relate to the characteristics of the groups invited, the nature of the invitation or decision aid, the precision and invasiveness of the test, the prevalence, severity and treatability of the disease screened for, or the rigor of the study designs and measures used to evaluate screening interventions. Trials of informed choice in screening may be particularly vulnerable to methodological bias. Traditionally, screening has been promoted by health professionals, so self report measures of screening attendance might be inaccurate. Furthermore, the consent process might select participants who are more likely to attend for screening [35]. For example, people refusing consent to participate in a screening trial may do so because they have no intention of attending for screening. In a trial of colorectal cancer screening, over 75% of general practice patients contacted chose not to consent to take part in the trial [34]. By contrast Krist and colleagues [32] recruited men to a prostate cancer trial taking place during a health examination that the men had already scheduled. Only 5% did not consent. In addition, there is evidence that questionnaires can alter research outcomes [36-38]; baseline questionnaires may prompt greater reflection on aspects of screening covered in the measures, thereby altering responses.

Given the uncertain effect of attempts to support informed choice and to evaluate its effects on screening uptake we have designed a rigorous pragmatic, population based randomized trial evaluating the effect on uptake of screening for type 2 diabetes of a validated invitation designed to inform the choice to attend. This trial protocol is designed to measure real screening decisions, in a primary care setting, unbiased by prior consent to study participation. This is the first trial of an informed choice decision aid for screening which includes all these design features.

### Screening for type 2 diabetes

Screening for type 2 diabetes and cardiovascular risk was chosen for study as type 2 diabetes is a condition for which behavior change (increasing physical activity, improving diet adherence to medication) is a major component of treatment [25], and for which at-risk population-based screening programmes are now being proposed and implemented [39-41]. Type 2 diabetes is a serious condition that is commonly undiagnosed until complications occur [42,43]. Existing evidence suggests that the adverse consequences of screening are likely to be limited [44,45] and that intensive treatment of clinically diagnosed patients is beneficial [24].

Uncertainties remain in relation to the overall cost-effectiveness of detection by screening and initiation of treatment earlier in the trajectory of the disease [43]. Evidence suggests that screening individuals at high risk of undiagnosed diabetes is most cost-effective [43,46], and that preventing complications arising from diabetes through behavior change in individuals in the early stages of the disease is more effective than treatments for those complications [47,48]. Patients at higher risk of undiagnosed diabetes can be identified using the Cambridge Risk Score [42,49-51], which can be used to select patients to invite for diabetes screening and to explore the impact of risk of undiagnosed diabetes on lifestyle choices.

### Study objective

The study objective is to estimate the impact upon attendance for diabetes screening of an informed choice invitation compared with a standard invitation. This is examined both overall and stratified by social deprivation. A secondary objective is to describe, amongst attenders, intention to change health-related behaviors if diabetes were subsequently detected. Intentions amongst attenders receiving the informed choice and standard invitations are compared.

The trial tests three principal hypotheses:

1. Attendance at screening for diabetes is lower following an informed choice compared with a standard invitation

2. There is an interaction between the type of invitation and social deprivation: attendance following an informed choice compared with a standard invitation is lower in those who are more rather than less socially deprived.

3. Amongst those who attend for screening, intentions to change behavior to reduce risks of complications in those subsequently diagnosed with diabetes are stronger following an informed choice invitation compared with a standard invitation

## Methods

### Design

**Figure 1:**
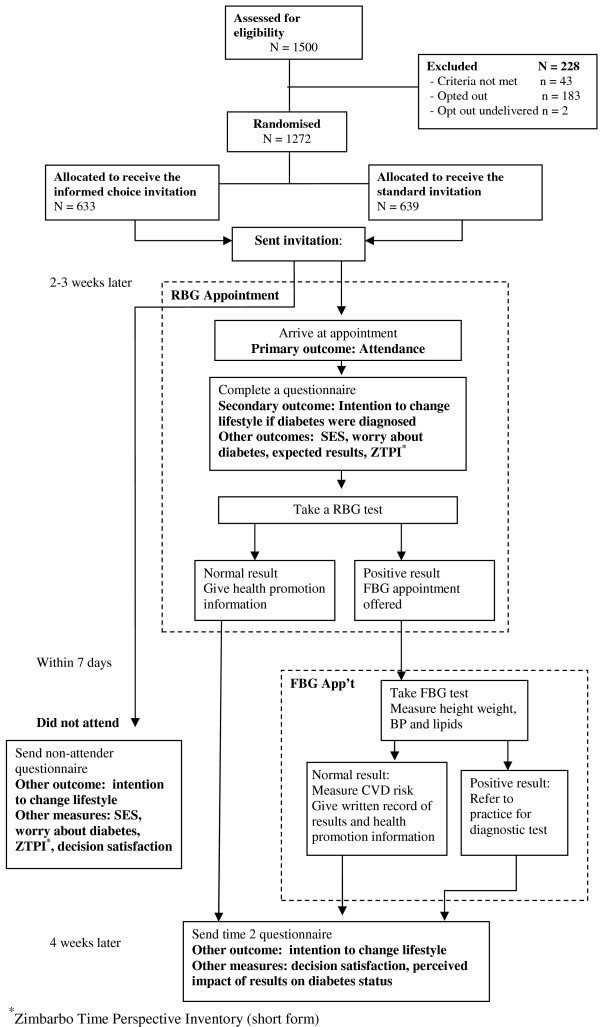
**Diagram of participant flow through the DICISION trial**.

The study design and participant recruitment (completed July 2007) is shown in figure 1. DICISION is a randomized controlled trial testing the impact of an invitation designed to facilitate informed choice (referred to as the informed choice invitation) on attendance for type 2 diabetes screening. The group that receives the informed choice invitation is compared to a group that receives an invitation typical of current practice (referred to as the standard invitation). Randomization of individuals by household clusters is undertaken from a central site following stratification by cluster size and general practice. The trial is set in four general practices and participants are recruited from practice lists. The informed choice invitation is designed to be replicable in a primary care setting using minimal resources. The design protects the primary end point by measuring attendance before providing further information about the trial or collecting questionnaire data that may have intervention effects. The trial is managed jointly between Kings College London and the University of Cambridge. COREC approval has been given (REC: Cambridgeshire 1: 06/Q0104/17, 05^th ^May, 2006), and R&D approval has been obtained from Greater Peterborough PCP, and Suffolk West PCT.

### Practice recruitment

The study is conducted in four general practices recruited from Cambridgeshire and surrounding counties, including two with above average area deprivation scores (in or above the middle quintile of the Index of Multiple Deprivation 2004). The practices are selected using a combination of information on practice participation in the Wellcome funded ADDITION trial (ISRCTN86769081), deprivation scores, and local interest in participation in research.

14 practices are selected for initial contact by letter, to invite participation and request a meeting between the practice team and the research team. At this meeting, research and clinical procedures are outlined, and members of the practice team are provided with a written summary of the study and a Research Information Sheet for Practices [52]. Before making a decision they are asked to discuss the study with the rest of the practice team and inform the trial co-ordinator of their decision within one month. Four study practices and two reserve practices are needed to ensure a sufficient sample size.

### Participant recruitment

The sample is selected from practice registers. It includes participants without known diabetes and in the top 25% of risk of having prevalent undiagnosed diabetes defined by a validated risk score. The Cambridge Risk Score [42] has been validated as a pre-screening instrument for the identification of those at increased risk of having prevalent undiagnosed diabetes [42,49-51,53]. For example, Griffin and colleagues [42] reported an area of 80% under a receiver-operating characteristic curve. Risk scores are obtained from a MIQUEST READ code based search of routinely-held practice data (weight, height, BMI, age, gender, antihypertensive and steroid medication). Risk scores have been calculated for over 150,000 people in practices in Cambridgeshire and surrounding counties participating in the ADDITION trial [54]. It is estimated that sufficient practice data are recorded to enable risk score calculations to be undertaken for around 70% of patients.

### Exclusion criteria

Patients meeting the following criteria are excluded from the trial:

i. Diagnosed with diabetes since the medical record search was performed.

ii. Pregnant or breast-feeding.

iii. Have a psychotic illness, such as schizophrenia, hypomania, major depression, manic depression.

iv. Have an illness with a likely prognosis of less than one year to death.

### Consent process

Potential participants are sent a letter from the practice with which they are registered informing them that the practice is going to undertake screening for diabetes as part of a research trial, and asking them to opt out if they do not wish to be invited. Those wishing to opt out are asked to return a freepost card to the practice.

When individuals attend for screening they are given the trial "patient information sheet" and then asked for consent to complete two questionnaires and for screening test results to be seen by researchers. For those who do not attend, information is given and consent obtained at the time of sending a questionnaire by post.

### Development and evaluation of the intervention materials

#### Standard invitation

The standard invitation is modeled on invitations commonly used to invite people for diabetes and coronary heart disease screening [11,55]. The text states that the participant is being offered screening because they might have a higher chance of developing type 2 diabetes, and that diabetes has serious long-term consequences (see additional file 1). An appointment date and time is also included, as well as instructions for rearranging or cancelling the appointment. The text is designed to be comprehensible to those with a reading age of 11 or above (Flesch Reading Ease score was 71.52, Flesch-Kinaid Grade level = 6.34).

#### Informed choice invitation

The informed choice invitation contains the same information as the standard invitation, plus information about risk of diabetes and its complications, and the likelihood of individual positive and negative consequences of attending for screening (see additional file 2). Participants are encouraged to make a choice that reflects their values by prompting evaluation of consequences, and asking them to record their decision to attend or not. Flesch Reading Ease score = 72.88 and Flesch-Kinaid Grade level = 5.76, indicating that the invitation should be readable by those with a reading age of 11 or above. Study materials and documents described in this protocol are available from the corresponding author on request.

##### Providing information about diabetes risk and consequences of screening

This section is developed from the UK General Medical Council (GMC) guidelines for providing sufficient information when gaining patient consent [56]. The invitation begins with an emphasis on patient choice; "*Screening for diabetes. It's your decision*", and is followed by the information given in the standard invitation. An invitation to think about possible benefits and harms before deciding whether to attend is then followed by an explanation of diabetes and the screening procedure. This is followed by an explanation of the different possible results and their meaning. An outline of how diabetes is treated with lifestyle change and pills is presented, followed by a presentation of the size of the possible individual benefits and harms of attending for screening and participating in treatment for diabetes were it to be subsequently diagnosed. All this information is given in words, numbers and graphically displayed using pie charts.

##### Encouraging participants to make a value consistent choice

A decisional balance sheet [57] is used to facilitate value-action consistency. It comprises an instruction to participants to write down the positive and negative consequences of attending diabetes screening for them, followed by their decision as to whether to attend their appointment or not, or to think more about whether or not they want to go for screening.

### Validation of the informed choice invitation

A randomized controlled analogue study was conducted among a volunteer sample of the general public in order to establish whether this invitation results in higher rates of informed choice than the standard invitation. Informed choice was assessed using a multidimensional measure of informed choice [27], which defines such choice as a behavior consistent with values in the context of appropriate knowledge [26,27].

Levels of informed choice were significantly higher following receipt of an informed choice invitation compared to the standard invitation; immediately after receipt (49.6% versus 7.2%; difference = 42.5% (95% CI: 33.7% to 50.3%); χ^2 ^= 72.922(1), p < 0.001) and two weeks later (42.9% versus 11.2%; difference = 31.7% (95% CI: 22.5% to 40.5%); χ^2 ^= 41.121(1), p < 0.001). This effect reflected increased knowledge of the benefits and costs of screening which was low, but not changes in attitude or intention to attend for screening which were highly positive [58].

### Procedure

#### Recruitment and randomization

Each of the four practices run the MIQUEST READ search program on the practice medical records and supply the trial statistician with the results for patients with diabetes risk scores in the top quartile. Each patient is assigned an ID number and 350 are randomly selected (450 from the final practice). Practice staff check patients' medical records to exclude any patients meeting the exclusion criteria outlined above. The remaining patients are sent the letter informing them of the trial and giving them an opportunity to opt out of receiving an invitation. Returned opt out forms are collated by a member of practice staff, and the trial statistician is informed of the IDs of the excluded and opted out patients.

The estimated 1200 individuals remaining after exclusions and opt-outs are randomized from a central site, stratified by practice and by cluster size (the number of individuals eligible for randomization in a household after removing those choosing to opt-out or excluded by the practice). This ensures that all eligible individuals in a household are co-selected and co-randomized to the same arm.

Selection and randomization is performed by the trial statistician with access only to data needed to calculate the risk score and to stratify the randomization, and is independent of the trial co-ordination team and database.

Randomized participants are sent either an informed choice invitation or a standard invitation to a screening clinic conducted by a DICISION research nurse, taking place at the patients' practice. Letters are sent from the practice, and appointments are pre-assigned. Participants can contact the practice as normal to rearrange or cancel their appointments.

#### Data collection

Attendance is recorded by a research nurse upon the participant's arrival. The participant then reads a patient information sheet, the nurse answers questions about the study and written consent is obtained from the participant. Where consent is refused, participants retain the opportunity to take the screening test if they wish. Where consent is obtained, participants then complete a questionnaire containing the measures shown in table 1.

**Table 1:** Study measures

Measures	From practice records	At appointment, but before RBG test	4 weeks after appointment	If appointment not attended
**Attendance**		X		
**Questionnaire measures**				
Intention to change lifestyle if found to have diabetes (Intention 1)*		X		
Intention to change lifestyle (Intention 2)			X	X
Worry about getting diabetes [63]		X		X
Expected test results*		X		
Time Orientation (ZTPI – Short form [64])		X		X
Age, gender, ethnic group		X		X
Social deprivation indicators: qualifications, home and car ownership		X		X
Decision satisfaction [61]			X	X
Perceived impact of results on diabetes status*			X	
**Demographic variables**				
Postcode (to identify IMD 2007 score [60])	X			
Risk indicators: BMI, age, sex, prescribed antihypertensives and steroid medication	X			

* Attenders only

The nurse then explains the screening procedure and carries out a finger prick random capillary blood glucose (RBG) test using HemoCue B-glucose analyzer based on the glucose dehydrogenase reaction (HemoCue AB, Angelholm, Sweden). The stability of the analyses is checked daily and an external calibration with the quality assurance scheme is undertaken monthly. Participants with a RBG level of less than 5.5 mmol/L are given standardized basic lifestyle advice on health eating and exercise. Those with RBG level equal to or greater than 5.5 mmol/L are invited to return for a fasting blood glucose test, carried out by the research nurse. Where blood glucose is over 22 mmol/L the participant is referred to the practice for review that day.

The subsequent fasting blood glucose (FBG) appointment is offered as a necessary clinical duty of care to the participant. A finger prick blood test is conducted to measure fasting capillary blood glucose level and a full lipid profile. Height, weight, blood pressure and cardiovascular risk score ([59]; the Cardiac Risk Assessor software is available from Prof Paul Durrington, University of Manchester) are also measured. Patients are offered further standardized lifestyle advice. Those with FBG levels equal to or greater than 6.1 mmol/L are referred to the practice for diagnostic testing.

Four weeks after the RBG appointment, consenting attenders are sent a follow up questionnaire containing the measures listed in table 1. Questionnaires are returned to the trial co-ordinator.

If a person cancels or misses their appointment, their non-attendance is recorded and a member of the practice team sends a non-attender pack to the patient, consisting of a questionnaire, a patient information sheet, consent form and reply-paid envelope. If no response is received within 3 weeks, then a reminder letter and another non-attender pack is sent. Measures contained in the non-attender questionnaire are shown in table 1. As practices are responsible for the administration of appointments, occasionally a participant who misses their appointment without informing the practice (classified as a non-attender) may subsequently rearrange and attend another appointment (therefore change to attender status). These participants are included as attenders in an intention to treat analysis. However, it is possible that the non-attender questionnaire prompts attendance in this case, rather than the invitation. We do not expect this to occur frequently, or to vary by trial arm. We will, however, record the number of attenders who are sent a non-attender questionnaire, and supplementary analyses excluding these participants may be necessary.

### Participant safety

Data collection and clinical procedures are detailed in a written clinical protocol and are managed by a grade H research nurse. Research nurses have up to date Hepatitis B vaccination, are certified in providing Basic Life Support, and follow the joint Addenbrooke's and University of Cambridge policy for managing exposure to blood. Procedures for dealing with medical emergencies are followed when necessary.

#### Adverse events monitoring

A detailed adverse events protocol has been produced for the research nurses by the clinical coordinator (SPS), including potential adverse events, their management and reporting procedures. The definition of adverse events excludes abnormal study outcome results, which are dealt with in the clinical protocol. All adverse events are reported to the trial coordinator and to the principal investigator. A signed and dated report is logged in the Trial Management File. Any adverse event deemed to be a serious adverse event after consultation with the principal investigators is reported to the University and NHS ethics committees within 24 hours.

### Main trial outcomes and measures

#### Attendance (primary outcome)

The primary outcome among those randomized to receive an invitation to screening is attendance for diabetes screening as a proportion of those randomized. Attendance is defined as arrival at an appointment, regardless of subsequent participation or previous missed appointments. All other randomized participants are defined as non-attenders.

#### Social deprivation

The primary measure of social deprivation is the Index of Multiple Deprivation 2007 (IMD 2007[60]). The IMD 2007 is published by the UK Department for Communities and Local Government. Geographical areas (approximate population 1500 per area) are rated on seven indicators of deprivation: i) income, ii) employment, iii) health, deprivation & disability, iv) education, skills and training, v) barriers to housing and services, vi) crime, and vii) living environment. The scores are weighted, summed and transformed resulting in a score from 0–100 where an average score of above 50 represents the 10% most deprived areas in the UK. Patient postcodes are used to identify a deprivation score for where they live.

#### Attenders' intention to make lifestyle changes if found to have diabetes (secondary outcome; intention 1)

The secondary outcome among those attending for screening is intention to make lifestyle changes if subsequent tests were to show that they had diabetes. This measure is a behavioral expectation in response to a hypothetical scenario, measured at the screening appointment with a self completed questionnaire, prior to taking the diabetes test. It is the mean of three intention items relating to medication adherence, reducing fat intake and increasing physical activity. Items are rated on a 7 point rating scale. An example of one of the items is: "If the tests show that you definitely do have diabetes, how likely is it that you will increase the amount of physical activity that you do over the next 3 months" (1 = extremely unlikely – 7 = extremely likely).

#### Decision satisfaction

This is measured in the four-week follow up questionnaire sent to attenders. A three item measure assesses the extent to which the choice is considered to be a good one, satisfaction with the way in which the decision was made, and certainty that the decision made was the best one for them e.g. "How sure are you that the decision you made was the right one for you?" (1 = not at all – 7 = extremely). In an earlier study this was found to have good reliability [61].

#### Supplementary trial outcomes and measures

Six other measures were assessed:

##### Intention to make lifestyle changes (intention 2)

Attenders' and non-attenders' intentions to make lifestyle changes are assessed in the four-week follow up questionnaire for attenders, and in the non-attenders' questionnaire. This measure is a behavioral expectation of actual lifestyle change and is structured in the same way as the item described above, as Intention 1.

##### Multiple individual-level social deprivation index

A secondary measure of individually defined social deprivation is used in supplementary analyses of participants who respond to questionnaires. A 5-point scale giving scores ranging from zero to four is calculated from self reported demographic information [62]. A participant scores one point for each of the following criteria that apply to them: a) do not own a car, b) do not own their own home, c) do not have post-16 level educational qualifications, and d) do not have any educational qualifications.

##### Additional self report measures

Additional self report measures include worry about getting diabetes [63], present and future orientation, using a short form of the Zimbardo Time Perspective Inventory (ZTPI [64]), and for attenders only, expected results and perceived impact of results on diabetes status. Descriptions of the questionnaire measures and the times of measurement are summarized in table 1.

### Power

A sample size of 1200 participants ensures 90% power to detect a 10% difference in attendance between arms using a comparison between proportions with a chi-squared test at the 5% significance level. With a predicted average attendance of 65% [11], 780 attenders are expected. Responses from all 780 attenders to the initial questionnaire provides 80% power to detect a small effect size (0.2 sd standardized difference) between arms in the primary intention measure using a t-test at the 5% level of significance. Allowing for a 75% response rate at the second time-point amongst attenders, there is 80% power to detect 0.25 sd effect size between arms for the continuous measures of intention and decision satisfaction. Assuming a small mean cluster size of 1.2 participants per randomized household and a conservatively high intracluster correlation of 0.8, secondary analyses allowing for clustering maintain 90% power to detect a 10% difference in attendance and 80% power to detect a 0.25 sd effect size amongst attenders.

### Statistical analysis

The chi-squared test is used to compare intention to treat attendance and other proportions between arms. The t-test is used to compare means of attenders' intention and other continuous measures between arms. Linear and logistic regressions are used to assess moderators using interaction terms where moderator variables are maintained as continuous. All analyses are two-tailed and assessed at the 5% level of significance. For statistically significant analyses, Donner's z-test for proportions and the linear mixed effects model for continuous outcomes are used in secondary sensitivity analysis accounting for clustering, assessing whether randomization by household affects the conclusions.

## Discussion

DICISION is a rigorously designed pragmatic population based clinical trial of an invitation to type 2 diabetes screening, designed to facilitate informed choice. The trial tests the hypotheses that knowledge of the uncertain personal benefits and potential harms of diabetes screening results in lower attendance, but amongst those attending, a higher likelihood of changing behavior to reduce complications of diabetes, were it to be subsequently diagnosed. We are also testing the hypothesis that facilitating informed choice reduces diabetes screening attendance to a greater extent in those who are socially deprived. Due to report in January 2009, this trial will provide a robust estimate of the impact of a policy of facilitating patient informed choice in diabetes screening in a primary care setting.

The trial design is pragmatic in that it closely reflects a screening programme in primary care up until attendance is measured, ensuring that the primary outcome cannot be influenced by prior measurement. Participants are selected from practice registers on the basis of routine data signifying increased risk of diabetes. Invitation letters are sent from participants' GPs and screening takes place at the surgery. An opt-out method of seeking consent to measure attendance is used. An opt-in method of consent to participate in the remainder of the trial is used after the primary outcome is measured. Providing significant information before attendance could affect the decision to attend, particularly in the comparison group, and so diminish the effect of the informed choice invitation [35]. Using the same procedure for obtaining consent in another screening study resulted in 4% opting out [65], suggesting that external validity is not compromised by adopting this method of consent.

The primary outcome is objectively measured; all participants can be identified as either an attender or a non-attender. Questionnaire data are collected after the primary outcome is recorded as evidence suggests that questionnaires may alter research outcomes [36-38]. In addition, the questionnaires are brief to maximize response rates.

An evidence-based invitation was developed from GMC guidelines [56] to provide up-to-date information about diabetes screening. Invitation text and layout were developed using evidence-based risk communication recommendations [66]. The effectiveness of the intervention was tested in a randomized controlled analogue study. The results showed that the informed choice invitation compared with a standard invitation was effective at achieving higher rates of informed choice, due largely to its increasing knowledge [58].

This is the first trial, to our knowledge, assessing the impact of facilitating informed choices for screening that uses a validated informed choice invitation and objectively measures screening uptake, whilst controlling for the methodological biases described above. As a result it is not always clear whether differences in findings between studies relate to the characteristics of the disease screened for, or to differences in trial methods. Screening programmes differ in the precision and invasiveness of the test offered, as well as the prevalence, severity and treatability of the disease for which screened is being offered. For example the potential harms of diabetes are less tangible than those for prostate cancer screening, for which treatment can lead to incontinence or impotence [67]. Trials of decision aids for prostate cancer screening tend to show lower uptake following the use of decision aids aimed at facilitating informed choices [32,68-70] although not always [71]. Trials of informed choice in colorectal screening, prenatal diagnosis and breast cancer screening more often found no difference in screening intention or uptake [29].

There are drawbacks to validating the intervention in an analogue study and evaluating its impact in a clinical trial. For example, in the analogue study all participants were observed to read their invitations, whereas in the clinical trial we can only be certain that participants are sent invitations. Invitations may not be received or, if received, may not be read properly. There are also other limitations. We used an area level index of social deprivation in individuals. As patients from the same practice live in geographically similar areas, an area level index is likely to have less variation than an individual level measure. It is used, however, to enable an intention to treat analysis of moderator effects on uptake. Nonetheless, this trial addresses some key limitations of previous trials through using previously validated invitations and an opt-out consent procedure in a real world setting.

The generalizability of the findings from this trial to other screening programmes is unknown. The harms that can arise from screening for diabetes are generally not considered serious and are described in the invitations as comprising worry prior to an appointment and false reassurance following a "screen negative" test result. In contrast, undergoing other screening tests can entail physical harms that include disability and even death e.g. colonoscopy [72]. Evidence of the impact of knowledge about such potential harms and the uncertain limited individual benefit of screening is mixed [29,73]. The extent to which the results of the current trial can be extrapolated to these other contexts will await results from similar trials to the one described in this protocol.

## Competing interests

The authors declare that they have no competing interests.

## Authors' contributions

TMM, ALK, SG, ATP, SS, MP are Principal Investigators; ATP is the trial statistician; EM is the trial coordinator; IK is the Pilot Study coordinator and contributed to protocol development; SPS is the clinical coordinator for the trial. All authors read and approved the final manuscript. TMM is the paper guarantor.

## Pre-publication history

The pre-publication history for this paper can be accessed here:



## Supplementary Material

Additional file 1 **The standard invitation.** Click here for file

Additional file 2 **The invitation designed to facilitate informed choice.** Click here for file
