# Supplementary material for: Impact of an informed choice invitation on uptake of screening for diabetes in primary care (DICISION): trial protocol
Source: BMC Public Health. 2009 Feb 20;9:63. doi: 10.1186/1471-2458-9-63 (PMC2666721; doi:10.1186/1471-2458-9-63)
Supplement: Additional file 1 — The standard invitation. [file 1471-2458-9-63-S1.pdf]

***<practice headed paper>***

<Patient EMIS number>
<Patient name>
<Patient address>

<space for handwritten date>

Dear <patient>

***Screening for diabetes***
Your surgery is offering screening for Type 2 diabetes. People who are at increased risk of developing diabetes are being invited to come for a simple, finger-prick blood test. Details in your GP health records suggest that you might have a higher chance of developing it. These details include your age, gender, medication, weight, and family history of diabetes.

***What is Type 2 diabetes?***
About 9 in every 100 people in the UK between 40 and 70 have Type 2 diabetes. Unfortunately, half of them don't know it. Most people with Type 2 diabetes usually feel well in the early stages of the disease. But this can hide the fact that diabetes is a serious disease that causes long-term problems.

***What now?***
*If you want to come for screening.*
A screening appointment has already been arranged for:

-----------------------------------------------------------------------------------------------

If you can't make this time, please contact the surgery for a time that is better for you. If you need interpreting services please let us know at least a week before your appointment.

*If you do not want to come for screening.*
Please contact the surgery to say that you do not want the appointment.

Yours sincerely,

<practice contact name>
