# Supplementary material for: Impact of an informed choice invitation on uptake of screening for diabetes in primary care (DICISION): trial protocol
Source: BMC Public Health. 2009 Feb 20;9:63. doi: 10.1186/1471-2458-9-63 (PMC2666721; doi:10.1186/1471-2458-9-63)
Supplement: Additional file 2 — The invitation designed to facilitate informed choice. [file 1471-2458-9-63-S2.pdf]

<practice headed paper>

<Patient EMIS number>
<Patient name>
<Patient address>

<space for handwritten date>

Dear <patient>

Screening for diabetes. It's your decision.
Your surgery is offering screening for Type 2 diabetes. People who are at increased risk of developing diabetes are being invited to come for a simple, finger-prick blood test. Details in your GP health records suggest that you might have a higher chance of developing it. These details include your age, gender, medication, weight, and family history of diabetes.

Before you decide whether to come for screening you might want to think about the possible benefits and possible harms.

What is Type 2 diabetes?
About 9 in every 100 people in the UK between 40 and 70 have Type 2 diabetes. Unfortunately, half of them don't know it. Most people with Type 2 diabetes usually feel well in the early stages of the disease. But this can hide the fact that diabetes is a serious disease that causes long-term problems.

The main long-term problems from diabetes are heart disease and strokes. Diabetes can cause blindness as well. It can also lead to kidney failure, amputations, and impotence. People with diabetes often have shorter lives.

What does diabetes screening involve?
Screening takes place at the surgery. A finger-prick blood test is taken. This checks the sugar (glucose) level in your blood. It shows whether you might have diabetes. After about 10 minutes, a nurse will tell you your result. Your whole visit should take about 30 minutes.

What will I be told and what does it mean?
If you come for screening you will be told one of two results.

1) *Told* ***"you do not have diabetes now***". This means that you almost certainly don't have diabetes. If 100 people had the test, about 63 would get this result. However, screening is not completely perfect. Of the 63 people getting this result, about 3 would have diabetes but the test would miss it.

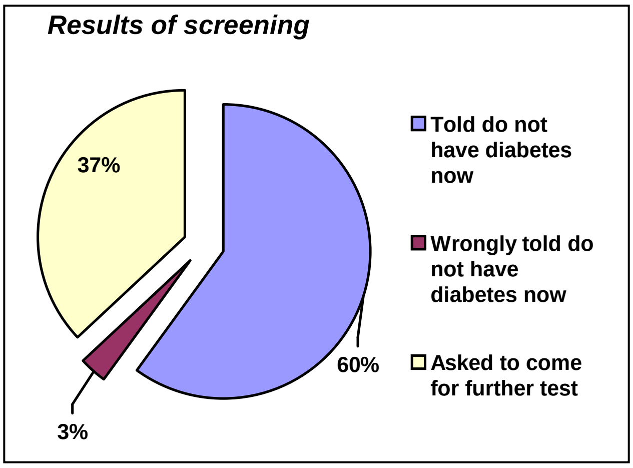

2) *Told "**you may have diabetes now**".*
This means that you might have diabetes. If 100 people had the test, about 37 would get this result. These people will need to come back to the surgery for another finger-prick blood test to find out if they have diabetes. Their cholesterol levels would be tested at the same time

How is diabetes treated?
Treating diabetes involves changes in lifestyle. These changes include eating healthily and taking regular exercise. They can also include taking medicines to reduce blood sugar, blood pressure, and blood cholesterol levels.

What are the possible benefits of coming for screening?

- A few people with diabetes benefit from early treatment in the first 10 years and avoid serious problems from their diabetes.
- Most people do not benefit from early treatment in the first 10 years of having the disease. This is because most people will not develop serious problems from their diabetes that soon. We can't be sure of later benefits of early treatment.

To estimate the benefits of early treatment for diabetes, imagine 100 people with diabetes 10 years after finding out they had it:

- **Without early treatment for diabetes**

16 people will have had a heart attack.
3 people will be blind in one eye.

- **With early treatment for diabetes**

12 people will have had a heart attack.
2 people will be blind in one eye.

- **This means that because of early treatment for diabetes**

4 people will avoid a heart attack.
1 person will avoid going blind in one eye.

Early treatment is effective. However, most people who would suffer these problems still will, even with early treatment.

How many people benefit from early diabetes treatment in the first 10 years?

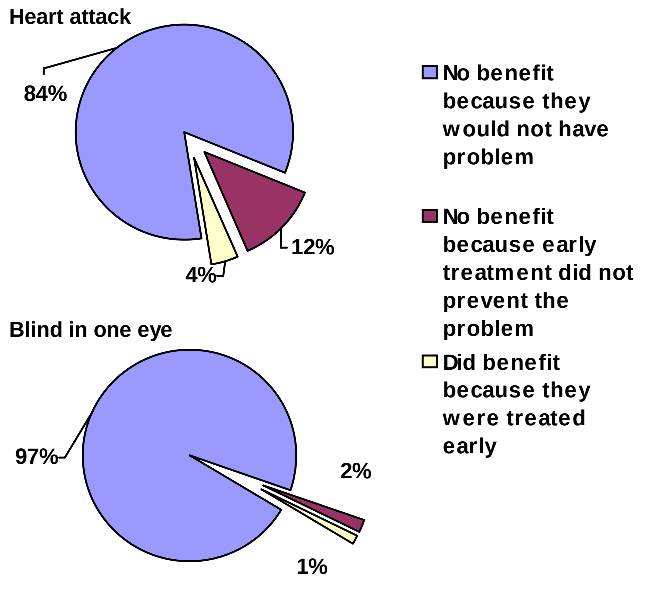

What are the possible harms of coming for screening?

- Coming for screening and being told "you do not have diabetes now" can make some people feel wrongly that they have no risk at all of developing diabetes. This can make them feel they don't need to take exercise or eat a healthy diet.
- Coming for screening makes some people worry. They find it difficult to relax or get to sleep, especially if they are asked to come for another test.

Deciding whether to come for screening.
When making your decision, it might help you to think about the good and bad things that could happen if you are screened for diabetes. Spend a few minutes thinking about having screening for diabetes. Please list all the good and bad things that go through your mind. Please underline the things that are most important to you.

**Good things from screening for diabetes.**

**Bad things from screening for diabetes.**

Having thought about the good and bad things of screening for me, my choice is:

- ❑ To go for screening.
- ❑ Not to go for screening.
- ❑ To think more about whether I do want to go for screening.

What now?
If you want to come for screening.
A screening appointment has already been arranged for:

If you can't make this time, please contact the surgery for a time that is better for you. If you need interpreting services please let us know at least a week before your appointment.

If you do not want to go for screening, please contact the surgery to say that you do not want the appointment.

Yours sincerely,

<practice contact name>
